# Inositol Phosphates and Retroviral Assembly: A Cellular Perspective

**DOI:** 10.3390/v13122516

**Published:** 2021-12-15

**Authors:** Clifton L. Ricaña, Robert A. Dick

**Affiliations:** Department of Molecular Biology and Genetics, Cornell University, Ithaca, NY 14853, USA; cr476@cornell.edu

**Keywords:** HIV, lentivirus, inositol hexakisphosphate, InsP6, IP6, InsP5, IP5, inositol phosphate metabolism, review

## Abstract

Understanding the molecular mechanisms of retroviral assembly has been a decades-long endeavor. With the recent discovery of inositol hexakisphosphate (IP6) acting as an assembly co-factor for human immunodeficiency virus (HIV), great strides have been made in retroviral research. In this review, the enzymatic pathways to synthesize and metabolize inositol phosphates (IPs) relevant to retroviral assembly are discussed. The functions of these enzymes and IPs are outlined in the context of the cellular biology important for retroviruses. Lastly, the recent advances in understanding the role of IPs in retroviral biology are surveyed.

## 1. Retroviral Assembly and Inositol Phosphates

**Figure 1:**
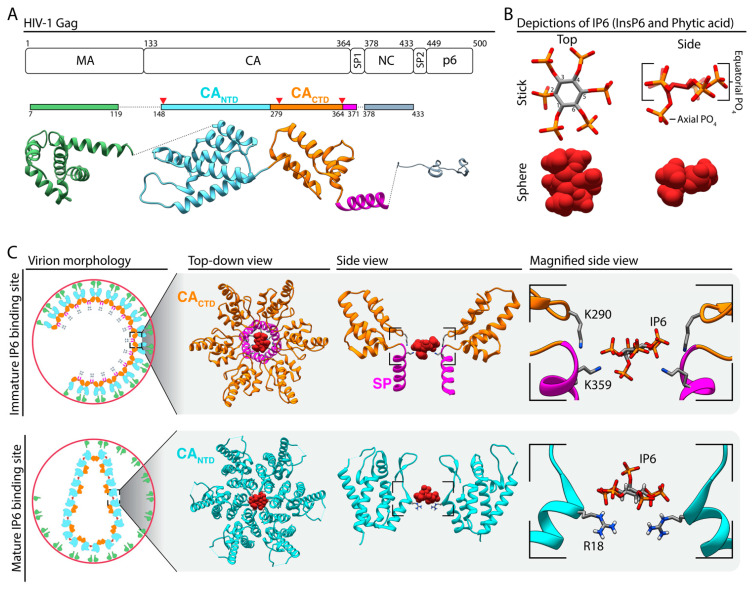
HIV-1 Gag protein, IP6, and virus assembly with IP6. (**A**) Illustration of the HIV-1 Gag protein with amino acid numbering and domains. The colored bars represent the span of amino acids in the structure depicted which was prepared from the following PDBs; MA (1HIW [8]), CA-SP1 (5L93 [9]), and NC (1NFS [10]). Red triangles mark the location of known IP6 binding sites. (**B**) IP6 stick and sphere depictions are used here. Numbering of the carbon and the corresponding PO_4_ position. The five equatorial PO_4′_s (1’,3’,4’,5’,6’) and the one axial PO_4_ (2’) are labeled. (**C**) Virion morphology of immature (top) and mature (bottom) HIV-1 virus following Gag cleavage and formation of the CA lattice, and the characterized IP6 binding sites for each. (Top) The immature IP6 binding site at the CA_CTD_ (orange) SP (purple) hexamer interface; image adapted from the crystal structure 6BHR [14]. Side view has two front and two back CA_CTD_-SP1 molecules removed to allow for a clear view of the IP6 binding site. Magnified view of IP6 interacting with lysine side chains K290 and K359 (Gag amino acid numbering). (Bottom) The mature IP6 binding site at the CA_NTD_ hexamer interface; image adapted from crystal structure 6BHS [14]. Side view has two front and two back CA_NTD_ molecules removed to allow for a clear view of the IP6 binding site. Magnified view of IP6 interacting with arginine side chain R18 (CA amino acid numbering). HIV Gag is used here as an example. For a detailed comparison of known and predicted IP6 binding sites in other retroviruses see [15].

For most retroviruses, virus particles are assembled at the plasma membrane. In this process, about 2000 molecules of the structural protein, called Gag (Figure 1A), interact with each other, with the genomic viral RNA (gRNA), and with the inner leaflet of the cellular plasma membrane to create a curved, hexameric lattice that bulges outward from the cell [1,2,3]. About 100 molecules of the Gag–Pol fusion protein also are incorporated into the lattice, thus bringing into the nascent virus the Pol proteins PR (protease, which cleaves Gag for maturation), RT (reverse transcriptase, which converts the viral RNA to DNA), and IN (integrase, which integrates the viral DNA into the host genome). This immature lattice pushes the membrane out, incorporating the viral envelope (Env) proteins that are embedded in the membrane [4]. The immature virus particle is pinched off from the cell by cellular ESCRT proteins and thus released into the surrounding medium [5,6,7]. The viral protease (PR) then cleaves Gag into its constituent domains, yielding the canonical mature proteins MA (membrane associated) [8], CA (capsid) [9], NC (nucleocapsid) [10], and others, depending on the virus species. The immature Gag lattice is broken down during proteolysis, and about 1000–1500 CA molecules then form a new lattice, also based on hexameric subunits [1]. The mature CA protein comprises two separately folded parts, here called CA_NTD_ and CA_CTD_ (N- and C-terminal domains, respectively). While maturation is required for infectivity, in cultured cells, Gag expressed by itself can bud to form immature virus-like particles, without the need for Pol, Env, or gRNA. Schematics of the retroviral genome, immature, and mature virion morphologies using Human Immunodeficiency Virus (HIV) as a model, and the inositol hexakisphosphate (IP6) binding sites are presented in Figure 1.

Virion assembly consists of an intricate balance of viral and host factors that promote Gag multimerization. Here, using primarily HIV-1 as the example (here called HIV for simplicity), we focus on the critical regions of Gag that form the base unit of the immature viral lattice, the hexamer. The six molecules of Gag are held together in large part by a six-helix bundle (6HB), with each helix being formed by the last few residues of the CA_CTD_ plus most of the 14-amino acid residue “spacer” domain, called SP1 [4]. The lattice of these hexamers is stabilized by CA_NTD_-CA_NTD_ dimer and trimer interactions between hexamers, CA_CTD_-CA_CTD_ dimer interactions, and NC interactions with the gRNA. Recently, the structure of the MA domain in the immature lattice was determined from virus-like particles (VLPs) produced from cells transfected with full-length HIV with an active site mutation in PR to prevent maturation [11]. The MA domain forms a hexameric lattice of trimers corresponding to the hexamer formed by the CA portion of Gag distal (toward the center of the virion) to MA [11,12]. The MA hexamer is held together by MA_NTD_–MA_NTD_ interactions between trimers. The trimeric MA provides stable contact points between hexamers [11]. The final immature hexameric Gag lattice is incomplete, i.e., has “gaps” [3,13].

The transition from an immature hexameric lattice to a mature one is mediated by sequential cleavage of Gag by PR [4]. The last cleavage steps separate MA from CA–SP1 and then SP1 from CA. The cleavage of SP1 likely causes a loss of six-helix bundle stability while the removal of MA causes a β-hairpin to form at the N-terminus of CA and promotes the folding of the more compact mature CA hexamer lattice [16]. This mature lattice resembles a fullerene cone and protects the gRNA and viral enzymatic proteins once the outer envelope is shed upon fusion with the target cell. Recently, MA has also been shown to undergo conformational changes during maturation [11]. The MA lattice in immature virions is significantly different than the MA lattice in mature virions. The hexamer of MA trimers shifts to a more tightly packed lattice creating larger regions of the viral membrane not occupied by MA in the mature virion. This restructuring is presumed to allow for Env conformational changes and clustering of many Env trimers in preparation to bind host receptor CD4 [17,18,19].

Cryo-Electron Tomography (cryo-ET) and subtomogram averaging (SA) of authentic immature virions have yielded high-resolution structures of the Gag lattice [9,20]. Much of the work characterizing structure and interactions at the atomic level has utilized in vitro assembly of purified Gag protein or Gag derivatives with early work reviewed by Bush and Vogt [21] and more recent work reported by the Briggs lab for HIV, Murine Leukemia Virus (MLV), and Mason-Pfizer monkey virus (MPMV) and the Schur/Dick labs for Equine Infectious Anemia Virus (EIAV) and Rous Sarcoma Virus (RSV) [11,22,23,24,25,26]. The first report of immature in vitro assembly with a purified protein was that for the alpharetrovirus RSV, which was found to require a truncated Gag construct (ΔMBDΔPR, i.e., deleted the membrane-binding domain and the PR domain), a nucleic acid at least 16 nucleotides in length, and mildly acidic conditions (pH 6.5) [27]. Other early studies focused on the betaretrovirus MPMV, for which the formation of spherical VLPs requires basic conditions (pH 8.8), but for the formation of immature tubular assemblies, requires a truncated construct (CANC) [28,29]. Initially for HIV, in vitro assembly of immature VLPs was found to be similar to that for RSV: most of the MA domain had to be removed for the formation of the immature lattice, and a short nucleic acid was required [30]. Attempts to extend these results to an intact Gag molecule (but still missing the short C-terminal p6 domain, here simply called HIV Gag) led to the formation of tiny, clearly biologically irrelevant particles about 25–30 nm in diameter [30].

In vitro assembly of purified CA protein into a mature lattice in most cases proved to occur only under highly non-physiological conditions. For example, RSV can assemble under acidic conditions (pH 4.9) and 500 mM NaPO_4_ [31], while HIV assembled efficiently at 2.5 M NaCl [32,33]. Furthermore, for HIV, the VLPs are overwhelmingly tubular, with only rare instances of cone-shaped particles resembling those in authentic budded virus particles. No in vitro conditions for mature MPMV assembly have been described.

In 1999, Campbell and Rein showed that adding tiny amounts of a rabbit reticulocyte lysate to an assembly reaction with full-length HIV Gag promoted the formation of VLPs closely resembling authentic immature virions [30]. In a follow-up study, the same group demonstrated that the active components in the cellular extract were inositol phosphates (IPs), specifically IP6 (I(1,2,3,4,5,6)P_6_, also called InsP6 or phytate) and inositol pentakisphosphate (I(1,3,4,5,6,)P_5_, abbreviated here as IP5) [34] (Figure 1B). In mammalian cells, steady-state levels of both IP6 and IP5 range from 10–100 μM [35,36,37,38,39]. Knowing that IP6 promotes in vitro assembly of full-length HIV Gag, but was not needed for Gag^ΔMA^ (here ∆MA refers to an internal MA deletion of amino acids 16–99 first described in [40,41]; due to the persistence of this annotation in the literature we will use ∆MA to refer to ∆16–99 as well unless otherwise noted), Campbell et al. assumed that IP6 must interact with the MA domain [34]. One hypothesis was that this highly charged small molecule mimics the polar head group of phosphatidylinositol 4,5-bisphophate PI(4,5)P_2_, which was known to play a role in Gag membrane interactions [42,43,44]. Consistent with this hypothesis, foot-printing studies showed that IP6 protects MA residues (as well as NC residues) from chemical modification [45]. However, the exact binding site for IP6 and the mechanism underlying its effects on in vitro assembly remained elusive [45], and indeed it was suggested that IP6 might be playing an artifactual role in in vitro assembly [42,43,44]. In 2016, however, John Briggs’ group showed that authentic VLPs from tissue culture had a density coordinated by a ring of positively charged lysines in the CA-SP1 6HB [9].

Almost two decades after the discovery that IP6 stimulates in vitro assembly of HIV Gag, a team led by Robert Dick revisited this subject [14]. IP6, and to a lesser extent IP5, dramatically stimulated assembly of the truncated Gag protein CA–SP1–NC to yield immature spherical VLPs. This protein does not contain the MA domain, thus excluding MA as a site of action. IP6 was also able to stimulate CA–SP1 assembly into immature-like structures. Further delineating the site of IP6 activity on assembly, IP6 stimulated CA_CTD_-SP1 to form flat crystals which were solved by x-ray crystallography by the Pornillos lab. This structure showed IP6 is coordinated by two rings of six lysines (K290 & K359) at the CA_CTD_ six-helix bundle interface (Figure 1C). Moreover, IP6 mapped directly to the density identified earlier by the Briggs group in native virions.

Dick et al. also demonstrated that IP6 stimulates mature HIV CA assembly [14]. Under physiological buffer conditions and in the absence of IP6, CA does not assemble. In the presence of IP6, CA assembles into abundant conical core-like structures. Crystal structures showed that IP6 interacts directly with a ring of six arginine residues (R18) in helix one of the CA_NTD_ hexamer interface (Figure 1C). In further support of IP6′s role in retroviral assembly, Leo James’s group also demonstrated that IP6, and to a lesser extent IP5, interacts with the R18 ring in mature CA assemblies and stabilizes the core [46]. This allows for more complete reverse transcription of gRNA compared to non-IP6-stabilized mature CA assemblies [46].

These in vitro assembly studies were corroborated in cell culture by knocking out (KO) the gene for inositol pentakisphosphate 2-kinase (IPPK), the enzyme that phosphorylates IP5 to yield IP6 [14]. Using CRISPR/Cas9 and 293FT cells, a cloned KO line was generated and tested for the ability to support HIV assembly. The KO cells were found to be severely defective in producing infectious virus particles compared to the parent 293FT cells [14].

## 2. Biosynthesis of Higher-Order IPs in Cells

**Figure 2:**
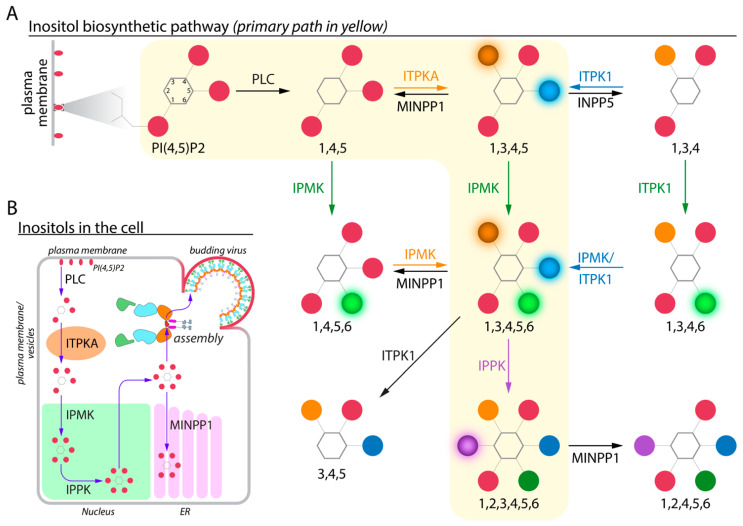
Partial inositol biosynthesis pathway of primary relevance to IP6 production. (**A**) Starting with the hydrolysis of the inositol head group from the plasma membrane-enriched phospholipid PI(4,5)P_2_, IP6 synthesis proceeds with the addition of phosphates to the IP3 molecule. The primary path leading to IP6 is highlighted in yellow. Enzymes are colored corresponding to the phosphate addition. Enzymes in black text represent de-phosphorylation steps. (**B**) The most commonly described IP pathway in cells and the dominant location of action. Labeling around and in the depicted cell corresponds to the organelle. After leaving the nuclear space, IP6 is utilized by HIV-1 Gag for immature virus assembly and subsequent budding. Alternatively, IP6 can be dephosphorylated by MINPP1 in the ER. IP names are simplified here to show only the positions which are phosphorylated.

**Figure 3:**
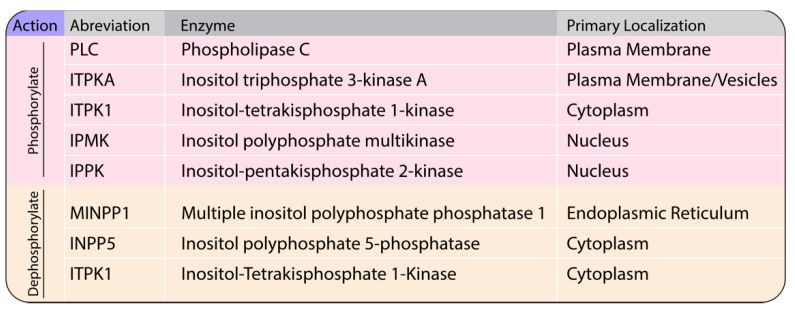
Action, abbreviations, enzymes, and primary location of action in cells.

IP6 is a hexagonal carbon ring (myo-inositol) with all six hydroxyl groups occupied by phosphates. IP6 and other higher-order IPs act as phosphate stores and as versatile signaling molecules in plant, yeast, and animal cells, but not in bacteria. Seminal findings and techniques used in IP research have been reviewed by Shears in the introductory chapter of *Inositol Phosphates: Methods and Protocols* [47]. Much of the early research focused on the IP6 content in plants, specifically agricultural feed. IPs, specifically IP5, were first shown in animals with their discovery in erythrocytes of birds and turtles [48]. Interest in IP research in animals was jump-started by Michael Berridge’s group, with the discovery of I(1,4,5)P_3_ signaling leading to Ca^2+^ release from the endoplasmic reticulum via phospholipase C (PLC) hydrolysis of PI(4,5)P_2_ in the plasma membrane [49]. With the use of the yeast model system, John York’s group and others over the years dissected the higher-order IP synthesis pathways as well as the role of IPs in cellular signaling, among many other functions [47,50,51,52,53,54,55,56]. A simplified IP pathway with the key enzymes that act to phosphorylate (kinases) and dephosphorylate (phosphatases) is shown in Figure 2. Enzyme names, abbreviations, and organelle of action are listed in Figure 3.

The IP synthesis pathway is complex and not fully resolved. As IP6 and IP5 are the relevant higher-order IPs for HIV assembly, the most important enzymes for their synthesis and metabolism are addressed here. The only human enzyme known to synthesize IP6 is inositol pentakisphosphate 2-kinase (IPPK), which converts IP5 to IP6 by adding one phosphate group. IP5 is synthesized via pathways that are not fully resolved. The primary enzyme for IP5 is thought to be inositol polyphosphate multikinase (IPMK), which phosphorylates IP4, but inositol tetrakisphosphate kinase-1 (ITPK1) may also play a role, although its pathway is unresolved. IPMK phosphorylates myo-inositol at positions 3, 5, and 6 (leading to I(1,4,5,6)P_4_, I(1,3,4,6)P_4_, I(1,3,4,5)P_4_, and finally I(1,3,4,5,6)P_5_, respectively). ITPK1 phosphorylates myo-inositol at positions 1 and 6 (I(3,4,5,6)P_4_, I(1,3,4,5)P_4_, and finally I(1,3,4,5,6)P_5_, respectively) [57,58,59,60]. Multiple inositol polyphosphate phosphatase-1 (MINPP1) can remove the 3-position phosphate from higher-order IPs [61,62,63,64]. MINPP1 converts I(1,2,3,4,5,6)P_6_ to I(1,2,4,5,6)P_5_, I(1,3,4,5,6)P_5_ to I(1,4,5,6)P_4_, and I(1,3,4,5)P_4_ to I(1,4,5)P_3_. These reactions are shown in Figure 2A.

The subcellular localization of these enzymes is important for understanding their function and their interactions with other cellular and viral proteins. Mammalian IPPK has a diverse localization profile dependent on cell type. IPPK is found throughout the cell in NRK 52E (normal rat kidney), COS7 (African green monkey kidney fibroblast-like), and H1299 (human lung carcinoma line derived from the lymph node) cells, but is most concentrated in the nucleus for all three cell lines [65]. Specifically, IPPK appears to be associated with euchromatin and nucleoli (Figure 2B), which correlates with known functions of IPPK and IP6 in the cell [65]. However, when over-expressed, IPPK also is found in discrete cytosolic foci corresponding to stress granules, as indicated by colocalization with mRNA, poly(A)-binding protein (PABP), and TIA-1-related protein (TIAR) markers [65]. In cancer cells such as A-431 (epidermoid carcinoma), U-2 OS (osteosarcoma), and U-251 MG (glioblastoma) cells, antibody staining for IPPK shows predominantly cytosolic expression [66,67,68,69]. Through measurement of transcript levels by RNA-seq, IPPK expression is detected in many blood cell types and, importantly for HIV, in CD4+ T-cells [66,67,68,70,71,72]. However, specific subcellular localization has not been determined. Consistent with its presence both in the nucleus and cytoplasm, IPPK has both non-canonical nuclear import and export signals (NLS and NES, respectively) [65]. Based on its highly charged nature and its relatively high concentration in cells, it has been speculated that IP6 as a freely soluble small molecule would disrupt cellular functions; thus, most IP6 is thought to be membrane- or protein-bound [50,51,73,74].

In contrast to the diverse cell-type-dependent localization of IPPK, the enzymes important for IP5 synthesis have distinct subcellular localizations (Figure 2B). Mammalian IPMK is found solely in the nucleus, also correlating with known functions of the enzyme and IP5 [75]. Specifically, a fluorescently tagged IPMK localizes to the nucleus in NRK 52E cells, with the NLS presumably being a C-terminal basic cluster in the enzyme [75]. Similar to that in IPPK, this NLS also appears to be non-canonical in structure [75]. In cancer cells such as A-431, U-2 OS, U-251 MG, and RH-30 (metastatic rhabdomyosarcoma) cells, antibody staining for IPMK is predominantly nucleoplasmic [66,67,68,76]. Mammalian ITPK1 is concentrated in mitochondria in U-2 OS cells [66,67,68,77]. Through measurements of transcript levels by RNA-seq, IPMK and ITPK1 expression are detected at low levels in many blood cell types, including CD4+ T-cells, but at moderate levels in granulocytes, specifically neutrophils [66,67,68,71,72,78,79]. However, subcellular localization in these HIV-relevant cells remains to be determined.

Finally, MINPP1 (the enzyme that breaks down IP6 and IP5) localizes to the endoplasmic reticulum (ER) lumen, for example in mouse embryonic fibroblasts (MEF) and NIH 3T3 cells (another mouse embryonic fibroblast line) [61,62,63,64], consistent with its conserved N-terminal ER signal peptide (Figure 2B). In H1299 cells, MINPP1 also is reported to be in the lumen of the lysosome. Together, these subcellular locales suggest that this enzyme has limited and tightly controlled access to higher-order IPs [80,81]. MINPP1 expression is detected in many blood cell types, including CD4+ T-cells, through transcript levels as quantified by RNA-seq, but subcellular localization has not been determined [66,67,68,71,72,82].

Just as localization is important for understanding function, the structure is equally important. Crystal structures of plant and mammalian IPPK show that the enzyme binds the first phosphate of IP5 to stabilize the closed structure required for the catalytic site to function. The structures also show that the mammalian orthologue, though conserved in amino acid sequence, has a unique zinc-binding site not found in the plant orthologue [83,84,85,86]. The crystal structure of the catalytic site human IPMK shows a constrained horseshoe-shaped catalytic site that uses Gln as the interacting amino acid residue, different from the plant orthologue, which uses Lys and Arg residues [87,88]. Furthermore, the unstructured regions of the protein apparently modulate ATP binding, and, thereby the kinase activity [89]. The crystal structure of ITPK1 from the parasite *Entamoeba histolytica*, which is highly conserved at the amino acid level to human ITPK1, shows an ATP grasp fold, with three phosphate-binding subsites allowing for multiple substrate specificities [90]. In addition to transferring phosphates between IPs, the crystal structure of human IPTK1 can transfer a phosphate from ATP to an IP via another binding pocket not found in plant or protozoan orthologues [91]. This transfer serves as a bridge between separate branches of the metabolic pathway: the metabolic cycle and the kinase/phosphatase pathways [91]. At the writing of this review, no structure for human MINPP1 has been reported. However, there are crystal structures for bacterial MINPPs that are packaged in outer-membrane vesicles, which are implicated in interacting with extracellular IP6 in the human colon, and are thermostable [92,93].

In the 81 years since IP5 was first found in avian erythrocytes, the functions discovered for higher-order IPs (IP5, IP6, and pyrophosphates, i.e., IP7) and the enzymes involved with their synthesis, metabolism, and degradation have expanded exponentially. Many review articles summarize the main findings over the decades [47,50,51,52,53,54,55,56,94,95,96,97].

## 3. Measuring Cellular and Viral IP Levels

Measuring IPs requires sophisticated techniques to separate not only the molecules with different numbers of phosphates but also the isomers of these IPs. The history and evolution of these techniques have been reviewed in more detail by others [47,55]. Here, we provide focus on those used in the context of a retroviral assembly, namely PAGE separation of IPs [39,98] and scintillation counting of tritium-labeled inositol derivatives [99]. Both are more sensitive and precise than indirect measurements such as phosphate bound to iron(III) [55].

The less intensive technique to measure cellular IPs utilizes PAGE separation [39]. In brief, cells are harvested, washed with PBS, and collected by low-speed centrifugation (pelleting). The cells are then incubated in 1 M perchloric acid (PA, pH 1) to extract IPs. Cell debris and precipitates are cleared from the extracted IPs by centrifugation. The supernatant is then incubated with TiO_2_ beads to bind and concentrate IPs. The beads are then washed with PA and incubated in 10% ammonium hydroxide (pH 10) to elute IPs. The eluent is then vacuum evaporated to concentrate the IPs and neutralize the ammonium hydroxide. After mixing with loading buffer, the IPs are separated via electrophoresis through a ~33% polyacrylamide/TBE gel and visualized via toluidine blue staining. This method separates IPs and nucleotides from each other but requires IP standards to identify the visible bands. PAGE separation requires the order of 10^7^ cells to obtain measurable levels of IPs, which is equivalent to about 500 ng of IP6 to form a visible band [39].

The more intensive, but more sensitive and precise, technique utilizes tritiated inositol ([H^3^]inositol) labeling of cells followed by high-performance liquid chromatography (HPLC) to measure fractions containing IPs [99]. In brief, cells are first seeded and allowed to adhere for 24 h. The medium is then aspirated, replaced with a medium containing [H^3^]inositol, and the cells are allowed to grow for three days. The medium is again aspirated, replaced with fresh [H^3^]inositol medium, and the cells incubated for an additional two days. After a total of five days of labeling, the cells are harvested, washed with PBS, and IPs extracted via acidic extraction buffer incubation as above. After neutralization, prepared samples are applied to a strong anion exchange (SAX) HPLC column. Collected fractions are then quantified by scintillation counting. To measure IPs incorporated into virions, cells are transfected with the proper viral plasmid components after the second [H^3^]inositol medium change and incubated for an additional three days [46]. Virions from virus-containing medium are pelleted through a 20% sucrose cushion and washed multiple times by resuspension in an inositol-free medium with subsequent pelleting through a 20% sucrose cushion. Viral pellets are then incubated in acidic extraction buffer and analyzed by HPLC and scintillation counting as above. While this method is time- and resource-intensive, the relative levels of labeled IPs can be more accurately measured from the small quantities found in viral pellets. For exact mass calculations, the mass of total cellular labeled IPs can be measured by PAGE separation as above, and the percent of the radioactivity in VLPs is used to back-calculate the mass incorporated in the VLPs [46].

## 4. Current Progress in Identifying the Roles of IP6 and IP5 in HIV Assembly

Since the publication of the papers implicating IP6 coordination by the HIV CA-SP1 6HB [14,46], much progress has been made in the field. In particular, the identification of the binding sites for IP6 on Gag has provided the platform for other advances. Identifying IP6 binding sites can be challenging since this very highly charged molecule is able to interact with diverse positively charged surfaces or pockets.

Alan Rein’s group initially provided evidence that IP6 binds to the HIV MA domain [45]. In their 2007 paper, they described trimerization of Gag monomers upon addition of IP6. IP6 stimulated trimerization was not observed when Gag was missing the MA domain. They hypothesized that IP6 could be a catalyst to promote Gag trimerization over dimerization, via IP6 interactions with MA. This is consistent with the MA trimerization previously observed via NMR [100,101]. MA trimers have been implicated in interactions with the Env cytoplasmic tail to facilitate the incorporation of Env into virions [102]. The Freed and Barklis labs recently demonstrated that the addition of IP6 to in vitro assemblies of purified MA induced trimerization and increased levels of the trimer-bound Env cytoplasmic tail [103]. However, they noted that IP6 could be mimicking the phospho-head group of PI(4,5)P_2_, which, when bound to MA, is reported to displace the bound gRNA, causing the myristate to ‘flip out’ and promote MA trimerization. While the exact role of IP6 in MA trimerization has not been fully parsed, a group led by Hasan DeMirci proposed genuine structural interactions between MA and IP6. Their work shows that the IP6 binding site on MA is adjacent to the PI(4,5)P_2_ binding site and that binding of the two small molecules may not be mutually exclusive [104,105]. However, a negatively charged density is not seen in the proposed binding site in the recently described MA immature and mature lattice structures of budded virions from cells [11]. Moreover, measurement of total IP6 in VLPs produced from cells demonstrated a 1:6 ratio of total Gag to IP6, i.e., one per CA hexamer [46]. Thus, further work will be necessary to determine whether the total intra-virion IP pool is split between MA and CA-SP1. The IP6 binding pocket described by the DeMirci group, thus, may represent an artifact due to the highly basic region of MA binding a highly negatively charged small molecule.

In 2018, Dick and colleagues demonstrated that IP6—and, to a lesser extent, IP5—coordinate positive lysine rings in the CA-SP1 6HB in the immature virion and positive arginine rings in the mature virion [14]. In addition, they demonstrated that the removal of IP6 from cells via KO of IPPK resulted in a significant decrease in infectious particle production. In 2019, the James group found that IP5 could also be involved in assembly through IPMK-KO in HEK293T and HeLa cells [106]. Furthermore, the small amount of virus that is released appears to incorporate residual IP6 or an elevated level of IP5 [106]. Additionally, the specific infectivity of the virions released from the KO cells was the same as from WT cells. In line with these data, the Johnson group later demonstrated that not only HIV but other primate lentiviruses require IP6 and IP5 [107]. Through complete depletion of these small molecules via exogenous expression of MINPP1 in the context of an IPPK-KO, HIV-infected cells were found unable to release any infectious virions. This defect was attributed to the inability of the virus to assemble or bud since full-length Gag was still produced at normal levels in the IPPK-KO/MINPP1^+^ cells. Additionally, it was demonstrated that virions package the required IP6 and/or IP5 and do not need these IPs to be present in target cells [106,107]. An overview of how IP6 and Gag may interact in the cell, as will be discussed in this section, is shown in Figure 2B.

While biochemistry and structural assays confirmed the binding of IP6 to the immature lattice [14,25] and basic virology techniques demonstrated the requirement for IP6, these methods cannot quantitatively elucidate the mechanistic effects during assembly. Dostálková et al. utilized the Ruml and Rumlová groups’ methods called Fast Assembly Inhibitor Test for HIV (FAITH) and Disassembly Inhibitor Test for HIV (DITH) to quantitively measure the efficiency and strength of IP6-induced immature and mature in vitro assembly [108]. They demonstrated that Gag (CA-SP1-NC) forms immature VLPs more efficiently in the presence of IP6 in a dose-dependent manner, with the highest efficiency at a 1:1 ratio of Gag hexamer to IP6, in accordance with findings from Dick et al. [14]. While the efficiency of mature assembly with CA-SP1-NC is not increased by the addition of IP6, VLPs are most stable, i.e., resistant to disassembly, at a sub-stoichiometric concentration of IP6 (1:0.12, CA-hexamer:IP6). However, it is difficult to assign a role for IP6 in this assay because both the immature and mature IP6 binding sites are present in the protein.

All-atom molecular dynamics (MD) simulations performed by Yu et al. in the Voth laboratory reveal a more detailed picture of these putative interactions of IP6 in the mature CA core [109]. This MD modeling shows the entry of IP6 into the hexamer pore and the initial contacts by R18 and K25-E29, as well as the entry of a second IP6 molecule. Moreover, in contrast with the hexamer, a modeled CA pentamer binds IP6 at a tilt so that the molecule is coordinated only by the R18 ring. This conclusion is further supported by an MD study by Xu et al., which shows that IP6 has 100% occupancy in the K25 pentamer ring [110]. The differences in IP6 interactions with hexamers and pentamers may have important biological consequences.

As discussed in the introduction, many of the mechanisms involved during assembly and maturation were elucidated in vitro. While separate in vitro models have been described for immature and mature assembly, a unified system for studying the transition between the two states was not available until very recently [111]: using the HIV Gag^ΔMA^ construct, a shortened gRNA containing the ψ packaging signal (required for incorporation of gRNA into the virion), and IP6, the Pornillos group was able to recapitulate the entire assembly–maturation process in vitro. In brief, bulk Gag^ΔMA^ is kept solubilized with 375 mM tartrate. Nucleation is induced with the addition of ψ gRNA, IP6, or both. Kinetics of assembly as indicated by turbidity is measured by light scattering at 320 nm and morphology by negative-stain EM over a time course. Using ψ gRNA results in immature VLPs with a small fraction of mature VLPs. The addition of IP6 synergistically accelerates the assembly kinetics and shifts the assembly to essentially all immature VLPs. Additionally, when purified HIV PR is subsequently introduced into the assembly reaction the morphology of particles is shifted to cones or tubes with a mature lattice, but only when ψ gRNA and IP6 are also present in the reaction mix.

Although IP6 promotes and stabilizes Gag immature assembly, maturation inhibitors, and mutations that cause hyper-stabilization render virions unable to mature, making them non-infectious. How does HIV utilize IP6 for stabilization, but remain pliable enough for subsequent disassembly and maturation? The James group addressed this question in cultured cells by showing that VLPs produced from cells treated with the maturation inhibitors (MI) bevirimat (BVM) or PF-46396 (PF96) incorporate the same amount of IP6 as VLPs produced from untreated cells [112]. This result indicates that IP6 and these inhibitors do not compete for the same binding site. However, it is not clear how PF96 binds though without displacing IP6 given that its predicted binding site (based on resistance mutations) overlaps with the IP6 binding site (PMID:23144615). Mutations in Gag that abolish one of the two lysine rings (CA numbering K158A and K227A; or more accurately Gag numbering K290A and K359A) render the virus highly deficient in IP6 interaction, and MIs no longer restricted spread, suggesting that the likely destabilization of the hexamer caused by the lysine mutants was compensated for by the MIs [112]. Just as second-site mutations arise in patients using MI, the passage of lysine ring-mutated replication-competent virus also yielded a second-site mutation, specifically T8I (SP numbering T8; or Gag numbering T371) in the SP1 domain [112]. This amino acid residue is located at the C-terminal end of the 6HB and was originally identified after passaging of MI-resistant HIV clones in the absence of MI’s [112]. This mutation hyperstabilizes the six-helix bundle [112]. Importantly, T8I rescues the K158A mutation by recovering the incorporation of IP6 into VLPs, this would have the added effect of IP6 being present for mature core formation. Interestingly, the second MI, PF96 partially restores infectious particle production of K158A by also recovering IP6 incorporation [112]. Concurrently, the Bieniasz group separately recapitulated the findings of Mallery et al. by also recovering the T8I second-site mutation after the serial passage of IP6 binding deficient HIV (NL4-3) K359A virus in MT4 cells [113]. Additionally, Poston et al. demonstrated BVM induction of HIV-mNeonGreen^K359A^ fusion protein assemblies by tracking the formation of green puncta in live cells [113].

Much of the work describing the role of IP6 and IP5 in retroviral assembly utilized in vitro methods and model cell lines such as HEK293Ts. Importantly, however, the biologically relevant cellular targets for HIV are CD4^+^ T-cells and macrophages. Sowd and Aiken recently validated the role of IP in retroviral assembly using CRISPR/Cas9 IPPK and IPMK knockouts in the biologically relevant MT4 and CEM T-cell lines with replication-competent HIV [114]. Just as in HEK293Ts, the IPPK-KO cells had no IP6 but increased cellular levels of IP5. The IPMK-KO displayed residual IP6, but little to no IP5. These authors demonstrated slowed replication kinetics in both cell lines when both genes were knocked out. Under thin-section EM, they saw no increase in the amount of incompletely budded or tethered virions compared with WT cells, but only fewer overall particles. Additionally, there was an increase in total intracellular Gag levels in the KOs compared with WT cells. Together, these results indicate that the small amounts of virus that are assembled in KO cells are able to bud properly and that the slower replication kinetics, in this case, is in part due to the inability of Gag to assemble at the plasma membrane. The Aiken group also demonstrated decreased infectivity of the virions, which can be attributed to an increase in non-virion-associated CA, defects in Gag processing, or lower amounts of Gag–Pol in virions. They speculate that the defects in Gag processing may be due to the inability of Gag–Pol to form PR domain dimers. Finally, the group also demonstrated that the KO CEM cell line was less permissive to infection through an undetermined mechanism. Taken together, the published data indicate that across several cell lines, removal of IP6 causes a severe decrease in immature virus release, but when normalized for release, both IP5 and IP6 support similar levels of infectivity.

## 5. The Role of IPs in Post-Entry Steps

While higher-order IPs were initially shown to play a role in assembly and maturation, IP6 has also been found to be important for the early steps in the retroviral life cycle, namely reverse transcription. Fasciculation and elongation protein zeta-1 (FEZ1), which is a kinesin adaptor protein, has recently been shown by the Perilla group to be the strongest known binder of mature CA hexamer. Using in vitro binding assays, cellular mutagenesis of FEZ1, and all-atom MD simulations, Huang et al. showed that FEZ1 binds the mature CA core at the R18 pore to facilitate motor protein-directed trafficking to the nucleus [115]. FEZ1 uses multiple negatively charged regions to bind the positively charged R18 pore. Though IP6 sits at this pore to stabilize the ring of positively charged arginines, FEZ1 is able to also bind to CA tubes at concentrations up to 200 μM IP6. However, further increasing IP6 concentration competed for FEZ1 off the tube hexamer lattice and suggests that after viral fusion, too much IP6 can inhibit trafficking of the viral core to the nucleus.

The R18 ring is also implicated in facilitating dNTP import through the hexamer pore into the CA core for use in reverse transcription. Using MD simulations, mutagenesis, and infectivity assays, Xu et al. showed that IP6 at the R18 ring mediates this dNTP translocation [110], possibly via a charged gradient mechanism. Interestingly, when another negatively charged small molecule benzenehexacarboxylic acid (BHC or mellitic acid) is used in place of IP6, MD simulations show that coordination of dNTP import is significantly reduced, as BHC binds more tightly to the R18 ring, which would limit the intra-virion pool of dNTPs necessary for reverse transcription.

To validate the MD simulations in a biologically relevant context, the NL4-3 virus with the K25N mutation was tested in HeLa and GHOST cells in addition to peripheral blood mononuclear cells (PBMCs). K25N virus is non-infectious with the defect occurring at reverse transcription, as indicated by significantly reduced levels of early and late transcripts as well as by 2-LTR circles (a measure for nuclear entry). However, when an additional hyper-stabilizing mutation E45A was introduced into the K25N mutant, the double mutant still was non-infectious as a result of defects in reverse transcription. For context, the single mutation E45A virus is only 10–30-fold less infectious than WT, while retaining WT levels of early and late transcripts as well as 2-LTR circles.

In line with the need for a stabilized CA core for FEZ1 recruitment and dNTP import, work from Mallery et al. in 2018 demonstrated that stabilization of the CA core by IP6 promotes reverse transcription [46]. This result was interpreted as IP6 prevents Capsid disassembly, thus protecting the gRNA and the viral enzymatic proteins. The Sundquist and the Pornillos laboratories as well as the Aiken and Rousso laboratories further recapitulated the role of IP6 in stabilizing the CA core for reverse transcription, and they were able to elucidate the mechanisms in vitro. Christensen et al. completely reconstituted reverse transcription and integration in vitro [116]: using authentic HIV-1 virions permeabilized using melittin to form pores in the viral membrane and expose viral cores to various treatments, the Sundquist and Pornillos groups demonstrated that low and high concentrations of IP6 inhibit reverse transcription. Low concentrations led to unstable cores and high concentrations to hyper-stabilized cores, both of which were inhibitory for proper reverse transcription. These phenotypes corresponded to phenotypes seen earlier when using mutations that hypo- and hyper-stabilize cores or when treating cells with the drugs PF74 or GS-CA1 [117,118,119,120,121,122].

Additionally, using tomogram reconstructions under various conditions, these authors identified patches in the hexamer lattice where newly synthesized viral DNA was extruded out of the cores. This piece of evidence gives credence to the model in which CA cores uncoat in the nucleus, where the majority of dNTPs are located. Lastly, the group also recapitulated integration in vitro by adding a cell lysate to the endpoint of the reaction mixture. Through sequencing, the authors demonstrated that high IP6 levels inhibited integration into exogenous DNA, implying that hyper-stabilized cores prevent break down and exposure of viral gDNA to the exogenous cellular DNA. Jennings et al. corroborated the findings by Christensen et al. [123]. Moreover, Rankovic et al. demonstrated by atomic force microscopy three observed peaks correspond to mechanical perturbations during reverse transcription, which correlates with different stages of DNA synthesis, likely during the strand transfer process [124].

## 6. The Role of IPs in Retroviruses Other Than HIV

Dick et al. showed that EIAV assembly is stimulated by IP6, and they also corroborated the effect on simian immunodeficiency virus (SIV), feline immunodeficiency virus (FIV), and bovine immunodeficiency virus (BIV) [25]. High-resolution cryo-ET and subtomogram averaging of EIAV VLPs clearly demonstrated that the EIAV IP6 binding site is the same as that previously described for HIV. Mallery et al. also demonstrated that IP6 and IP5 are necessary for wild-type levels of virus release for HIV-2, SIV, and FIV [106]. In a detailed study of the conserved role of higher-order IPs in lentiviruses, Ricaña et al. demonstrated, using cell culture techniques in WT and CRISPR/Cas9 IPPK-KO 293FT, that IP6 and IP5 are absolutely required by primate lentiviruses (HIV-1 and SIVmac), but in the cell lines tested, act as enhancers for immature assembly in non-primate lentiviruses (FIV and EIAV). Furthermore, they showed that the betaretrovirus MPMV and gammaretrovirus MLV do not require either IP6 or IP5. Not only do the Gag proteins of these viruses lack the lysine residues that form the immature IP6 binding site, but IPPK-KO or exogenous expression of MINPP1 does not affect virus production. In support of these conclusions, Dostálková et al. also recently reported no IP6 effect on MLV or MPMV assembly using their FAITH and DITH techniques [125].

The alpharetrovirus RSV has been the workhorse of retrovirology; however, a detailed structure of the complete CA core has not been solved. Obr et al. recently demonstrated that IP6 acts as an assembly cofactor for mature RSV cores, but, interestingly, not for immature viruses [26], consistent with the absence of Lys residues at the positions found in lentiviruses (HIV residues K290 and K359). This conclusion is supported by an almost 100-fold decrease in infectious particle production when RSV is produced from 293FT IPPK-KO cells compared to the parent cell line. As discussed in the introduction, in vitro assembly of immature RSV can be stimulated by nucleic acid and slightly acidic conditions, while mature RSV can be stimulated by NaPO_4_. Importantly, the authors provide a detailed tomographic reconstruction of RSV CA cores that depicts inherent flexibility of mature IP6-stabilized hexamers. In the mature CA hexamer, the equatorial plane of IP6 is oriented perpendicular to the Lys-Arg rings (K17 and R21; equivalent to R18 for HIV-1). The perpendicular orientation of IP6 actually binds in the pore formed by K17 and R21, differing from how IP6 binds above the pore formed by R18 for HIV-1. The density at the centers of the pentamers could not be assigned to IP6 due to the limitations of the resolution achieved. However, it is likely IP6 given the relative purity of the assembly reactions. In the icosahedral assembly, the density at the pentamer pore likely corresponds to NaPO4, as it was the only negatively charged ion.

## 7. Future Directions for Studying IPs in the Context of Retroviral Infection

Despite rapid progress in uncovering the mechanistic role of IPs in the retroviral life cycle, many questions remain to be answered. One such problem that may reveal underlying mechanisms is determining the ratio of IPs in the total HIV virion as opposed to the mature CA core. Furthermore, determining whether the mature core has CA hexamers that are not stabilized by IP6 could illuminate FEZ1-capsid interactions, since FEZ1 might use these unoccupied binding sites and not need to compete off IP6. Lastly, knowing the exact ratio of IP6 to CA hexamers in mature cores might provide insight into how viral lattice maturation proceeds.

Secondly, understanding where IPs are incorporated into Gag assemblies in the cytoplasm would address many interesting unknowns, such as specific trafficking locales in the late stage of the retroviral life cycle. Since the highly charged and reactive nature of higher-order IPs restricts their localization to specific subcellular compartments or membranes [50,51,73,74], it is likely that Gag is trafficked to these subcellular locales as well; if so, this would reveal new interactions between viral proteins, IP6, and other cellular factors/proteins that would be potential targets for antivirals. For example, IPPK is localized in the perinuclear space of the cytoplasm. If the ribosomal complex transcribing Gag is bound to or in the vicinity of IPPK, nucleation could be hypothesized to occur at the perinuclear space, since the gRNA would also be there. Riley et al. described a fluorescent conjugate of IP5 (FAM-IP5) which can be taken up by H1229 tumor cells [126]. If this conjugate can substitute for IP6 in HIV assembly, one could theoretically track the incorporation of FAM-IP5 in the context of an IPPK-KO line containing FAM-IP5. Such an experiment could more closely track nucleation and transit of Gag assemblies from translation to the plasma membrane.

Thirdly, while there have been a few studies recapitulating in vitro results and model cell line data in biologically relevant T-cells and PBMC, more validation of newly revealed mechanisms described above needs to be performed in T-cells and macrophages. A full understanding of the role of IP6 in these cells could reveal potential avenues and efficacies for inhibitors or other targets. For example, AP-2 and clathrin mediate the restriction of HIV; perhaps binding of IP6 competes out AP-2 binding of Gag in CD4^+^ T-cells, preventing clathrin-mediated recycling of Gag from the membrane [127,128,129,130,131]. Because IPs and the enzymes for their synthesis and metabolism are important for immune responses [132,133,134,135], understanding how IPs regulate immune effectors such as interferon in the context of HIV infection may reveal new insights into the immune response. These last musings are highly speculative, but without doubt, a better understanding of higher IPs will lead to a deeper appreciation of the role of these small molecules in the retrovirus life cycle.

## Data Availability

Not applicable.
